# Valence stabilization of polyvalent ions during gamma irradiation of their aqueous solutions by sacrificial protection. III-Valence stabilization of Fe(II) ions by organic additives

**DOI:** 10.1007/s10967-013-2610-z

**Published:** 2013-09-22

**Authors:** M. F. Barakat, M. M. Abdel Hamid

**Affiliations:** 1 Nuclear and Radiological Control Authority, 3, Ahmed El-Zomor Str., Nasr City, P. O. Box 7551, Cairo, 11787 Egypt; 2 Hot Lab. Center, Atomic Energy Authority, Cairo, Egypt

**Keywords:** Valence stabilization, Water radiolysis, Protective effects, Extended irradiation, Competitive reactions, Radiolysis

## Abstract

Valence stabilization of polyvalent ions in gamma irradiated aqueous solutions is sometimes necessary in some chemical operations. In previous publications, valence stabilization of some polyvalent ions in solution upon gamma irradiation was achieved by using inorganic additives capable of interacting with the oxidizing or reducing species formed during water radiolysis. The results showed that the nature and duration of valence stabilization of Fe(II) depend on the concentration of the inorganic additives used. In the present work, a series of some organic additives has been used to investigate their capability in inducing valence stabilization of polyvalent iron ions, taken as an indicator, in aqueous acidic solutions when subjected to extended gamma irradiation. The results showed that the efficiency of valence stabilization depends on the amount and chemical structure of the organic additive used.

## Introduction

Protective effects against structural and ionic changes induced by traces of some chemicals during irradiation by X- or γ-radiations have been treated by several authors [1–5]. It was suggested that protection of irradiated systems containing aqueous polyvalent ions is a phenomenon that is greatly related to competitive reactions. Competition depends on the reaction rates and also on the equilibrium constant of the oxidation reduction reactions occurring in gamma irradiated aqueous systems containing polyvalent ions.

The protective effect of additives in some aqueous irradiated systems was observed long time ago. Fricke et al. [6] showed that addition of formic acid to X-irradiated aqueous acetic acid solution highly reduced the oxidation of acetic acid even if the concentration of the protective agent is 100 times lower than the concentration of acetic acid.

Other authors reported on the existence of protective effects in case of methylene blue decolorization by α rays, in presence of formic, malonic acids or gelatin [7]. Saturated compounds act more weakly than unsaturated compounds [8].

Stabilization of the oxidation states of certain polyvalent ions when present in a strong irradiation field is an important problem in applied radiation chemistry.

It is well known that in aqueous irradiated systems the nature of reactions between solutes and primary products of water radiolysis are different. The reactions occurring in presence of transition metal ions generally occur by electron transfer (Oxidation–Reductions reactions) while in systems containing organic solutes the reactions predominantly occur by hydrogen abstraction or addition reactions [9]. It is therefore possible to expect that in aqueous systems containing both transition metal ions and organic additives as solutes both type of reactions can occur. Many studies have been carried out to investigate the role of some inorganic additives in valence protection of some multivalent ions during radiolysis of their aqueous solutions. The results showed that valance stabilization continues for periods dependant on the concentration of the additive used and is most probably due to the competition reactions of the multivalent ions and the additives for the oxidizing or reducing species formed in the systems due to water radiolysis [10–14].

It is well known that aqueous Fe(II) solutions are rapidly oxidized under the effect of gamma radiations. The present work aims at investigating the possibility of protecting the divalent state of iron ions during extended gamma irradiation by using a series of some organic additives. The results showed that the efficiency of valence stabilization of Fe(II) during extended gamma irradiation depends on the amount and chemical structure of the organic additive used.

## Experimental

In the present work, extended gamma irradiation of aqueous acidic iron ions solutions in presence of different types of organic additives comprising alcohols, aldehydes or organic acids, has been undertaken. The effect of additive type and concentration on the prevailing reactions of the polyvalent ions in the irradiated systems has been particularly treated.

### Chemicals and materials

- Extra pure ferrous ammonium sulphate (FeSO_4_(NH_4_)_2_SO_4_·6H_2_O), ferrous sulphate (FeSO_4_·7H_2_O), ferric sulphate (Fe_2_(SO_4_)_3_·9H_2_O) were obtained from May and Baker (M & B) Co. LTD., and the British Drug Houses (B.D.H.) England.
- Chemically pure methanol, ethanol, n-propanol and *n*-butanol were obtained from Camberian Chemicals, England. Acetaldehyde, propionaldehyde and butyraldehyde were supplied from Prolabo Co., Paris. Formic acid, chemically pure 100 %, sp.g. 1.231 was also obtained from Prolabo, France, acetic acid for analysis, 99–100 % sp.g. 1.055–1.050 was obtained from Fein Chemie K–H. Kallies KG, Germany. Propionic acid, 99 % was obtained from B.D.H. Co. England. All these chemicals except alcohols were used without further purification. All alcohols were distilled twice over freshly ignited and cooled CaO.
- Analytical grade chemicals such as 1.10, phenanthroline (M.W. 180.21) were obtained from B.D.H. England. Sulphuric acid (98 %); sp.g. 1.84, hydrochloric acid (35–37 %), sp.g. 1.18, were also obtained from B.D.H. England.

All other chemicals were of the analytical grade reagents and were used without further purification.

### Equipments

All pH measurements were carried out using an Orion Research pH meter model 616 A digital ion analyser with a combined glass-calomel electrode

Spectrophotometric measurements, were carried out using a Shimadzu UV–Vis double beam spectrophotometer type UV-2l0A. Glass or quartz cells were used whenever necessary.

Potentiometric titrations were carried out using a Radiometer type PO_3_ pH meter with Pt and saturated calomel electrodes.

### Preparation of solutions

All solutions were prepared using double distilled water. The water was boiled then cooled and stored in stoppered glass flasks.

#### Preparation of iron solutions

*Preparation of Fe(II) solution* A.R. FeS0_4_·7H_2_O crystals were washed twice with double distilled water followed by A.R. acetone and then dried by heating at 50 °C for a few minutes. Exactly 2.7803 g of the purified ferrous sulphate were weighed and dissolved in about 300 ml of freshly boiled and cooled bidistilled water. The solution was quantitatively transferred to a 1 l flask together with 22.2 ml cone. H_2_SO_4_, after being diluted with bidistilled water in 400 ml water. The resultant solution was then completed to the mark to give ~0.01 N Fe(II) solution in 0.8 N H_2_SO_4_.

The exact ferrous ion concentrations was determined titrimetrically with a standard (exactly about 0.1 N) potassium permanganate solution prepared as described in detail elsewhere. The end point was detected potentiometrically

*Preparation of Fe(III) solution* About 0.01 N Fe(III) solution was prepared by dissolving 3.999 g Fe_2_(SO_4_)_3_·9H_2_O in hot bidistilled water. The resultant solution was filtered and the filtrate was introduced into a 1 l volumetric flask together with 22.2 ml conc. H_2_SO_4_ previously diluted to 400 ml and the resultant solution was then completed to the mark with double distilled water to give a solution containing 0.8 N H_2_SO_4_. The exact concentration of ferric ions in the solution was titrimetrically determined against a standard EDTA solution using tiron indicator at 40–50 °C. At the equivalence point the solution turned from green to yellow.

### Preparation and irradiation of samples

The irradiated samples were prepared by taking 5 ml of 10^−2^ M Fe^2+^ or Fe^3+^ in 0.8 N H_2_SO_4_ together with the necessary amounts of different organic additives and the solutions were completed to the mark in 50 ml volumetric flasks. The resultant solutions were introduced into glass irradiation tubes (15 cm long and 2.5 m diameter) provided with a neck 1 cm in diameter ending with a ground glass stopper.

Irradiation of samples was carried out using a Canadian Co^60^ gamma cell-220 for extended time periods. The irradiation dose rate of the gamma cell was around 0.43 KGy per hour. Samples were withdrawn from the irradiated solutions at intervals and were analyzed. The irradiation dose of the irradiator was occasionally checked by the well known ferrous sulphate method.

During irradiation care was always taken to keep the position of the irradiation tubes unchanged along the whole irradiation time.

#### Analysis of irradiated solution

The concentration of existing Fe(II) ions in the irradiated solutions was followed spectrophotometrically at intervals by measuring the absorbance of the orange red complex formed with 1,10-phenanthroline against a reagent blank at 510 nm [15]. The molar absorptivity is 1.16 × 10^4^ [10]. The unknown concentrations of iron in the analysed samples were determined by calibration curves constructed within the concentration range 1.0 × 10^−5^–1.5 × 10^−4 ^M of Fe(II).

### Reaction rate constants

In the present work, all reaction rate constants (*k*) in dm^3^ mol^−1^ s^−1^, were used from the work of Anbar and Neta [16, 17].

## Results and discussions

In air free aqueous irradiated systems the following highly reactive primary water radiolysis products are formed. The corresponding *G* values, at pH 7 are as follows [18]:

In presence of small amounts of oxygen, H and OH radicals are rapidly scavenged as follows:

2 $$ {\text{O}}_{2} + {\text{H}} \to {\text{HO}}_{2}^{ \bullet } \quad K = 2.1\; \times \;10^{10} $$

3 $$ {\text{O}}_{2} + {\text{e}}^{ - } {\text{aq}} \to {\text{O}}_{2}^{ \bullet - } \quad K = 1.9 \times 10^{10} $$

Consequently, in aerated aqueous systems the most important reaction is the attack of OH radicals and to a lesser extent the perhydroxyl radicals (${\text{HO}}_{ 2}^{ \bullet }$) and perhydroxide radical anions (${\text{O}}_{2}^{ \bullet - }$) on the prevailing species in the irradiated systems [19].

It has been reported before that a 10^−3 ^M Fe(II) acidic solution (0.08 N H_2_SO_4_) was completely oxidized when irradiated for about 2 h, using a gamma dose rate of 310 Gy/h i.e. after absorbing about 620 grays [13].

In the present work, valence stabilization of Fe(II) during extended gamma irradiation, in presence of different aliphatic organic acids, aldehydes or alcohols, has been investigated. Thus, increasing amounts of some aliphatic acids, aldehydes or alcohols were added to a certain concentration of Fe(II) ions in acidic aqueous solutions and the concentration of existing Fe(II) was followed spectrophotometrically during the continued irradiation of the systems. In the following sections the obtained results are discussed.

### Valence stabilization of Fe(II) ions during extended gamma irradiation in presence of organic acids

**Fig. 1:**
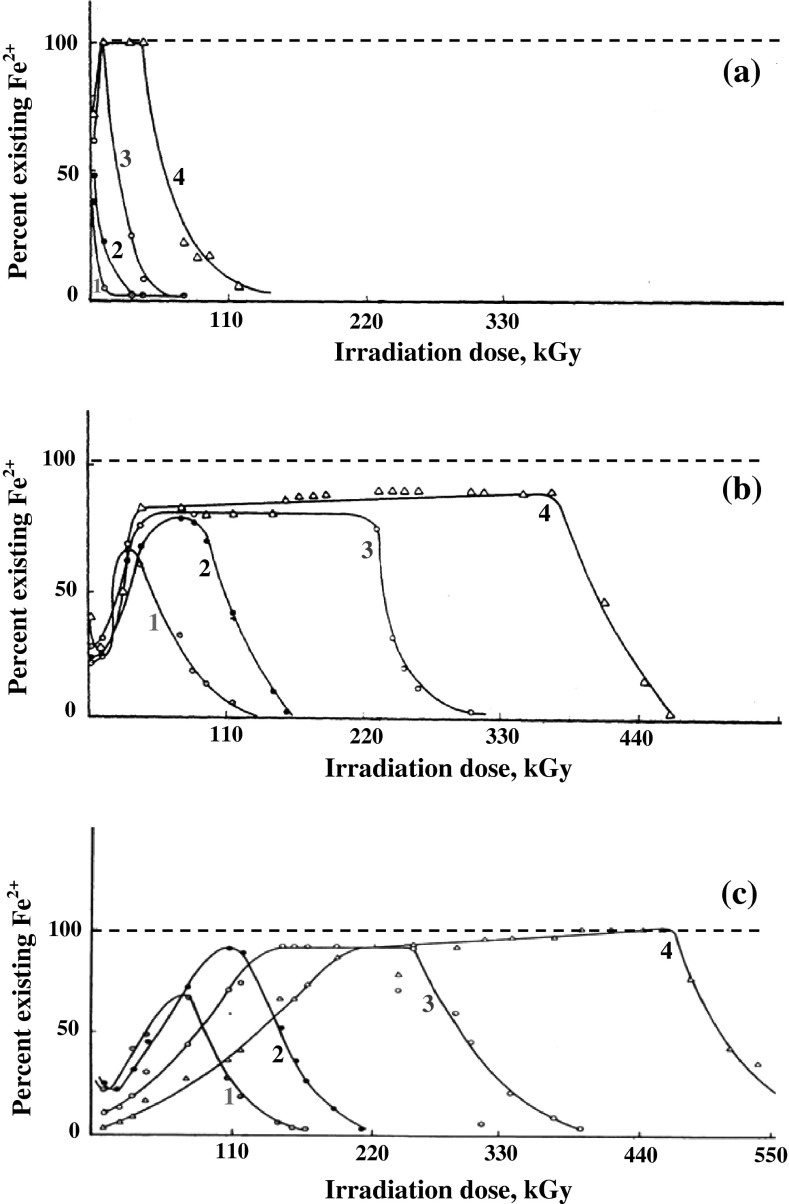
Percent existing Fe^2+^ in γ-irradiated 10^−3^ M Fe^2+^ solutions (0.08 N H_2_SO_4_) containing **a** Formic acid, **b** acetic acid **c** propionic acid; at various concentrations: *1—*1.6 × 10^−3^ M (*filled circle*) *2*—3.2 × 10^−3^ M (*times*) *3*—8.0 × 10^−3^ M (*open circle*) *4*—16.0 × 10^−3^ M (*open triangle*). *Dashed line* 100 % protection line, *solid* *line* actual protection line

In Fig. 1 the change of Fe(II) concentration during extended gamma irradiation of acidic Fe(II) solutions in presence of different organic acids is shown, by the solid lines which represent the actual protection lines. It could be observed that the effect of formic acid, being more easily oxidized to CO_2_ and water, is different from other fatty acids which have more than one carbon atom [9, p. 317]. In case of other acids valence protection of Fe(II) ions occurs in three steps. The first involves oxidation of Fe(II) to Fe(III), followed by gradual reduction of Fe(III) to Fe(II) again, the concentration of which remained almost constant during extended gamma irradiation, until finally at the end of the valence protection period, the concentration of Fe(II) ions gradually decreased being finally transformed to Fe(III) ions. That behaviour could be explained on the basis of competitive reactions occurring between H or OH radicals—formed in water radiolysis—and the polyvalent ion or the organic additive.

Thus, in presence of acetic or propionic acid additives, oxidation of Fe(II) ions occurs very rapidly by OH radicals as follows:

4 $$ {\text{Fe}}^{ 2+ } + \mathop {\text{O}}\limits^{ \bullet } {\text{H}} \to {\text{Fe}}^{ 3+ } + {\text{OH}}^{ - } \quad K = 1. 3\times 10^{ 9} $$

At that stage, the organic acid additive probably does not interfere due to their relatively lower reaction rates as compared to the reaction rate of OH with Fe(II).

5 $$ {\text{HCOOH}} + \mathop {\text{O}}\limits^{ \bullet } {\text{H}} \to {\text{H}}_{ 2} {\text{O}} + \mathop {\text{C}}\limits^{ \bullet } {\text{OOH}}\quad K = 1. 6\times 10^{ 8} $$

6 $$ {\text{CH}}_{ 3} {\text{COOH}} + \mathop {\text{O}}\limits^{ \bullet } {\text{H}} \to {\text{H}}_{ 2} {\text{O}} + \mathop {\text{C}}\limits^{ \bullet } {\text{H}}_{ 2} {\text{COOH}}\quad K = 1. 8\times 10^{ 8} $$

7 $$ {\text{C}}_{ 2} {\text{H}}_{ 5} {\text{COOH}} + \mathop {\text{O}}\limits^{ \bullet } {\text{H}} \to {\text{H}}_{ 2} {\text{O}} + {\text{CH}}_{ 3} \mathop {\text{C}}\limits^{ \bullet } {\text{HCOOH}}\quad K = 2.0 \times 10^{ 8} $$

When most of the iron ions are present in the Fe(III) state gradual reduction starts to take place by the action of H radicals as follows:

8 $$ {\text{Fe}}^{ 3+ } + {\text{H}}^{ \bullet } \to {\text{Fe}}^{ 2+ } + {\text{H}}^{ + } \quad K = 1. 3\times 10^{ 7} $$

Organic additives are only slightly capable of affecting the reduction reaction of Fe(III) by H radicals since competition between the organic acids and Fe(III) for H radicals occurs in favor of the H radical reaction with Fe(III), as could be deduced from the lower rate values of the following reactions:

9 $$ {\text{HCOOH}} + {\text{H}}^{ \bullet } \to {\text{ H}}_{ 2} + \mathop {\text{C}}\limits^{ \bullet } {\text{OOH}}\quad K = 2\times 10^{ 6} $$

10 $$ {\text{CH}}_{ 3} {\text{COOH}} + {\text{H}}^{ \bullet } \to {\text{H}}_{ 2} + \mathop {\text{C}}\limits^{ \bullet } {\text{H}}_{ 2} {\text{COOH}}\quad K = 1. 3\times 10^{ 5} $$

11 $$ {\text{CH}}_{ 3} {\text{CH}}_{ 2} {\text{COOH}} + {\text{H}}^{ \bullet } \to {\text{H}}_{ 2} + {\text{CH}}_{ 3} \mathop {\text{C}}\limits^{ \bullet } {\text{HCOOH}}\quad K = 3. 2\times 10^{ 6} $$

whereby, $$ k_{ 8} /k_{ 9} = { 38}, \, k_{ 8} /k_{ 10} = 6 1 5, \, k_{ 8} /k_{ 1 1} = { 13}. 5 $$

Moreover, organic carboxylate radicals, now present at higher concentrations, due to H and OH radicals reactions with acids by reactions 5–7 and 9–11 can also contribute to the reduction process of Fe(III) as follows [20]:

12 $$ \mathop {\text{C}}\limits^{ \bullet } {\text{OOH}} + {\text{Fe}}^{ 3+ } \to {\text{Fe}}^{ 2+ } + {\text{CO}}_{ 2} + {\text{H}}^{+ } $$

13 $$ {\text{R}}\mathop {\text{C}}\limits^{ \bullet } {\text{HCOOH}} + {\text{Fe}}^{ 3+ } \to {\text{Fe}}^{ 2+ } + {\text{R}}\mathop {\text{C}}\limits^{ \bullet } {\text{HCOO}} + {\text{H}}^{ + } $$

This could be further clarified by referring to the standard reduction potentials [21] of the following reactions:

$$ \begin{aligned} 2 {\text{Fe}}^{ 3+ } + 2 {\text{ e}} \to 2
{\text{Fe}}^{ 2+ } \quad  E^{\text{o}}   & =+0. 77 {\text{ v}}
\\ {\text{CO}}_{ 2} + 2 {\text{H}}^{ + } + 2 {\text{e}} \to
{\text{HCOOH}}\quad  E^{\text{o}}   & = - 0. 20  {\text{ v}}
\\
\end{aligned} $$

Using these standard reduction potential values, it is possible to find out that the equilibrium constant of the reaction [22]

$$ 2 {\text{Fe}}^{ 3+ } + {\text{ HCOOH}} \to {\text{ 2 Fe}}^{ 2+ } + 2 {\text{H}}^{ + } + {\text{ CO}}_{ 2} $$

is equal to 7.6 × 10^32^ which shows that the reaction is very favorable.

At that stage, reduction of Fe(III) continues until most iron ions were reduced to Fe(II) ions.

It could also be observed in Fig. 1c that in presence of propionic acid, reduction of ferric ions occurred more slowly than in case of acetic acid. This is probably due to the fact that propionic acid competes more effectively for H radicals than acetic acid. This is further confirmed by the decrease in the rate of Fe(III) reduction when greater propionic acid concentrations were used .

At the end of Fe(III) reduction stage the concentration of the formed Fe(II) ions remained almost constant during continued gamma irradiation for durations depending on the amount of organic acid used. During that stage OH radicals actively interact with the organic acid existing in the medium (by reactions 5, 6, 7) being present in greater excess than Fe(II) ions. At the end of the valence protection stage, the organic acid concentration gradually decreases and consequently OH radicals gradually interact with Fe(II) ions until most irons are transformed to the trivalent state.

### Valence stabilization of Fe(II) ions during extended gamma irradiation in presence of aliphatic aldehydes

**Fig. 2:**
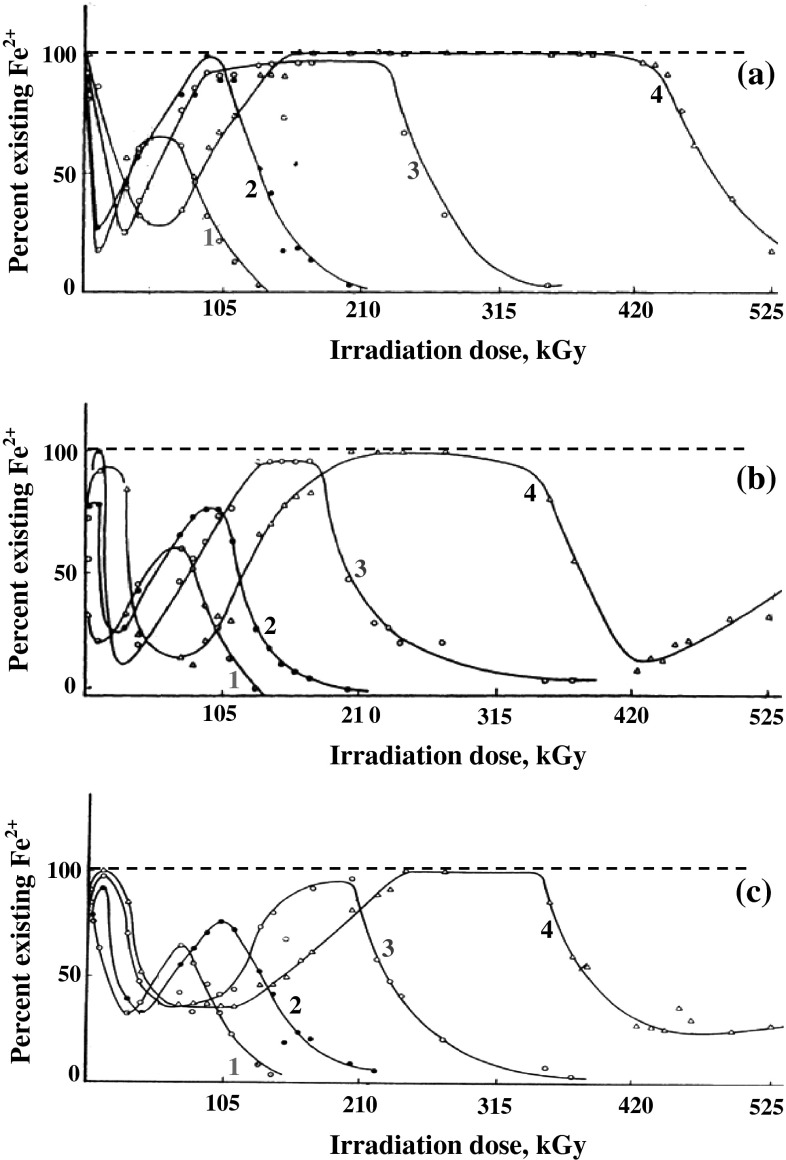
Percent existing Fe^2+^ in γ-irradiated 10^−3^ M Fe^2+^ solutions (0.08 N H_2_SO_4_) containing **a** Acetaldehyde, **b** Propionaldehyde, **c** Butyraldehyde; at various concentrations: *1*—1.6 × 10^−3^ M (*filled circle*) *2*—3.2 × 10^−3^ M (*times*) *3*—8.0 × 10^−3^ M (*open circle*) *4*—16.0 × 10^−3^ M (*open triangle*). *Dashed line* 100 % protection line, *solid* *line* actual protection line

In Fig. 2, the solid lines (the actual protection lines) shows the change of the concentration of Fe(II) ions during extended gamma irradiation of acidic Fe(II) solutions in presence of organic aldehydes. Two protection periods exist, the first of which occurs within 50–60 KGys while the second involves longer irradiation periods i.e. larger doses.

It has been reported before that at the beginning of irradiation Fe(II) ions are oxidized very rapidly in presence of aliphatic aldehydes and after a very short steady state Fe(III) is rapidly reduced to Fe(II) ions [12]. On continued irradiation Fe(II) ions survived until the end of the first protection period at the end of which rapid oxidation of Fe(II) to the trivalent state occured. The second protection period starts by the gradual reduction until most of Fe(III) is transformed to Fe(II), the concentration of which remained almost stable for durations depending on the aldehyde concentration used.

It is possible to assume that the stability of Fe(II) ions during the first protection period is very probably due to aldehydes acting as OH radical scavengers according to the following reactions:

14 $$ \begin{aligned} & {\text{CH}}_{ 3} {\text{CHO}} + \mathop {\text{O}}\limits^{ \bullet } {\text{H}} \to \mathop {\text{C}}\limits^{ \bullet } {\text{H}}_{ 2} {\text{CHO}} + {\text{H}}_{ 2} {\text{O}}\quad K = 7 { } \times { 1}0^{ 8} \\ & {\text{CH}}_{ 3} {\text{CH}}_{ 2} {\text{CHO}} + \mathop {\text{O}}\limits^{ \bullet } {\text{H}} \to {\text{CH}}_{ 3} \mathop {\text{C}}\limits^{ \bullet } {\text{HCHO}} + {\text{H}}_{ 2} {\text{O}} \\ \end{aligned} $$

15 $$ {\text{CH}}_{ 3} {\text{CH}}_{ 2} {\text{CH}}_{ 2} {\text{CHO}} + \mathop {\text{O}}\limits^{ \bullet } {\text{H}} \to {\text{CH}}_{ 3} {\text{CH}}_{ 2} \mathop {\text{C}}\limits^{ \bullet } {\text{HCHO}} + {\text{H}}_{ 2} {\text{O}}\quad K = { 2}. 3 { } \times { 1}0^{ 9} $$

While

4 $$ {\text{Fe}}^{ 2+ } + \mathop {\text{O}}\limits^{ \bullet } {\text{H}} \to {\text{Fe}}^{ 3+ } + {\text{OH}}^{ - } \quad K = 1. 3\times 10^{ 9} $$

Aldehydes being present in a great excess as compared to Fe(II) concentration can effectively remove OH radicals. During the first protection period continued gamma irradiation continuously changes aldehydes to the corresponding acids through their transformation to the hydrates followed by their interaction with H or OH radicals [19].

The formed organic radicals can easily change to the corresponding acids:

20 $$ 2 {\text{H}}\mathop {\text{C}}\limits^{ \bullet } ( {\text{OH)OH }} \to \, 2{\text{HCOOH }} + {\text{ H}}_{ 2} $$

21 $$ 2 {\text{CH}}_{ 3} \mathop {\text{C}}\limits^{ \bullet } ( {\text{OH)OH }} \to \, 2{\text{CH}}_{ 3} {\text{COOH }} + {\text{ H}}_{ 2} $$

22 $$ {\text{H}}\mathop {\text{C}}\limits^{ \bullet } ( {\text{OH)OH }} + {\text{ H}}_{ 2} {\text{O}}_{ 2} \, \to {\text{ HCOOH }} + {\text{ H}}_{ 2} {\text{O }} + \, \mathop {\text{O}}\limits^{ \bullet } {\text{H}} $$

At the same time, oxidation of Fe(II) by OH radicals is rendered ineffective. Oxidation of aldehydes to the corresponding acids can also occur by interaction of oxygen liberated from water radiolysis by the following reaction [23].

It is therefore possible to assume that aldehydes are continuously changed during irradiation to the corresponding acids.

At the beginning of the second protection period i.e. during the Fe(III) reduction by H radicals, aldehydes can enhance the Fe(III) reduction to Fe(II) being themselves changed to the corresponding acids [24, 25].

24 $$ 2 {\text{Fe}}^{ 3+ \, } + {\text{HCHO}}
\mathop{\longrightarrow}\limits^{{\rm H}_{2}{\rm O}} 2
{\text{Fe}}^{ 2+ \, } + {\text{ HCOOH }} + 2 {\text{H}}^{ + } $$

25 $$ 2 {\text{Fe}}^{ 3+ \, } + {\text{CH}}_{ 3} {\text{CHO}}
\mathop{\longrightarrow}\limits^{{\rm H}_{2}{\rm O}} 2
{\text{Fe}}^{ 2+ } + {\text{CH}}_{ 3} {\text{COOH}} + 2
{\text{H}}^{ + } $$

This could be further clarified by calculating the equilibrium constant of these reactions using the standard reduction potential values [22] of the half reactions involved. The equilibrium constant values obtained are 2.8 × 10^26^ and 1.4 × 10^30^ for reactions 24 and 25 respectively showing that these reaction are very favorable.

When all iron present is reduced to the divalent state, continued survival of Fe(II) ions during the second protection period very probably occurs by continued interaction of OH radicals with the existing acids. That continues until the complete exhaustion of the formed acids at the end of the second protection period.

### Valence stabilization of Fe(II) ions, during extended gamma irradiation, in presence of aliphatic alcohols

**Fig. 3:**
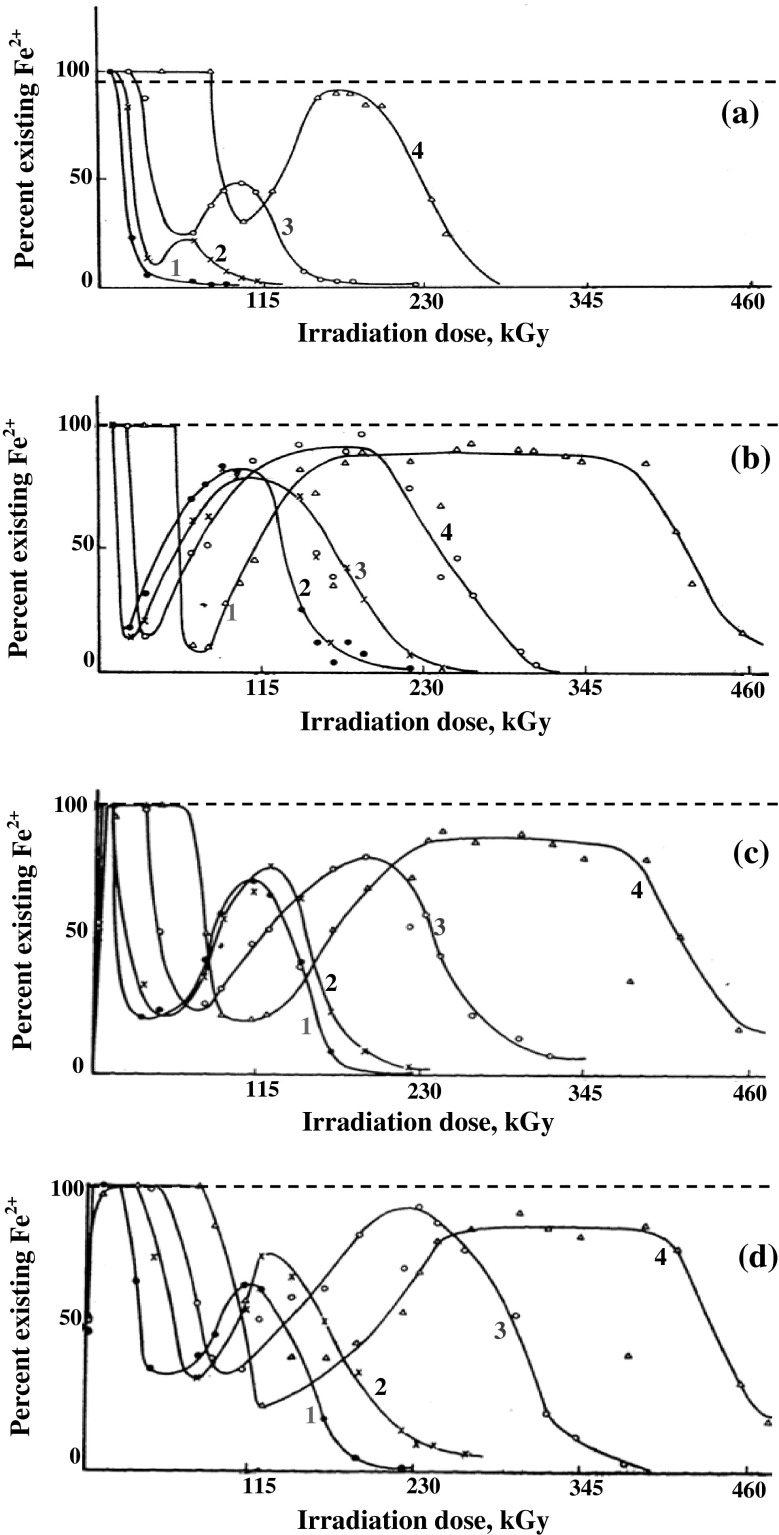
Percent existing Fe^2+^ in γ-irradiated 10^−3^ M Fe^2+^ solutions (0.08 N H_2_SO_4_) containing **a** Methanol, **b** Ethanol, **c** Propanol, **d** Butanol; at various concentrations: *1*—3.2 × 10^−3^ M (*filled circle*) *2*—4.8 × 10^−3^ M (*times*) *3*—8.0 × 10^−3^ M (*open circle*) *4*—14.0 × 10^−3^ M (*open triangle*). *Dashed line* 100 % protection line, *solid* *line* actual protection line

Varying amounts of some aliphatic alcohols were added to acidic Fe(II) solution to investigate the valence stability of Fe(II) ions during extended gamma irradiation of the solutions. The concentration of Fe(II) was followed spectrophotometrically and the results are given in Fig. 3. The solid lines represent the change of Fe(II) concentration during extended gamma irradiation periods.

It is possible to observe the existence of two protection periods separated by an oxidation then reduction stages. It is interesting to note that the first protection period in case of alcohols is much more developed than in case of aldehydes, while the second protection period is almost similar to that obtained in case of aldehydes and acid additives.

According to Broszkiewicz [11] at the beginning of irradiation of 10^−4 ^M Fe(II) in presence of aliphatic alcohols in 0.1 N H_2_SO_4_ at a dose rate of 2.97 × 10^16^ eV/ml min^−1^, organic peroxides are formed capable of oxidizing several Fe(II) ions per OH radical as follows [26]:

That probably explains the rapid oxidation of Fe(II) at the beginning of irradiation. This was followed by a steady state whereby after the absorption of 440 Grays reduction of the formed trivalent iron occurred gradually. In general, after about 1 h of irradiation complete reduction of iron ions to Fe(II) was observed.

In the present work, when the initial oxidation, steady state then reduction were over, divalent iron ions remained protected upon continued irradiation for periods dependant on the alcohol concentration used until the end of the first protection period. This probably shows that alcohols act as active scavengers for the oxidizing radicals during that period as follows:

26 $$ {\text{CH}}_{ 3} {\text{OH }} + \mathop {\text{O}}\limits^{
\bullet } {\text{H }} \to \, \mathop {\text{C}}\limits^{ \bullet }
{\text{H}_{2}} {\text{OH}}+ {\text{H}}_{ 2} {\text{O}}\quad K = 0.
5 3\times 10^{ 9} $$

27 $$ {\text{C}}_{ 2} {\text{H}}_{ 5} {\text{OH }} + \mathop {\text{O}}\limits^{ \bullet } {\text{H }} \to {\text{ CH}}_{ 3} \mathop {\text{C}}\limits^{ \bullet } {\text{HOH}} + {\text{H}}_{ 2} {\text{O}}\quad K = 0. 8 7\times 10^{ 9} $$

28 $$ {\text{C}}_{ 3} {\text{H}}_{ 7} {\text{OH }} + \mathop {\text{O}}\limits^{ \bullet } {\text{H }} \to {\text{ C}}_{ 2} {\text{H}}_{ 5} \mathop {\text{C}}\limits^{ \bullet } {\text{HOH }} + {\text{ H}}_{ 2} {\text{O}}\quad K = 0. 6 8\times 10^{ 9} $$

29 $$ {\text{C}}_{ 4} {\text{H}}_{ 9} {\text{OH }} + \mathop {\text{O}}\limits^{ \bullet } {\text{H }} \to {\text{C}}_{ 3} {\text{H}}_{ 7} \mathop {\text{C}}\limits^{ \bullet } {\text{HOH}} + {\text{H}}_{ 2} {\text{O}}\quad K = 4. 6\times 10^{ 9} $$

30 $$ {\text{Fe}}^{ 2+ } + \mathop {\text{O}}\limits^{ \bullet } {\text{H }} \to {\text{ Fe}}^{ 3+ } + {\text{OH}}^{ - } \quad K = 1. 3\times 10^{ 9} $$

Although the rate constants of the $\mathop {\text{O}}\limits^{ \bullet } {\text{H}}$ reactions with alcohols are slightly lower than the rate constant of $\mathop {\text{O}}\limits^{ \bullet } {\text{H}}$ reaction with Fe(II) yet, the greater concentration of the organic alcohols enhances their interaction with OH radicals and consequently the valence of Fe(II) ions remains rather stable. Moreover, alcohol radicals formed can further reduce any formed Fe(III) ions being themselves transformed to the corresponding aldehyde [27].

31 $$ {\text{RCH}}_{ 2} {\text{OH }} + {\text{ OH }} \to {\text{ R}}\mathop {\text{C}}\limits^{ \bullet } {\text{HOH}} + {\text{ H}}_{ 2} {\text{O}} $$

32 $$ {\text{R}}\mathop {\text{C}}\limits^{ \bullet } {\text{HOH}} + {\text{OH }} \to {\text{ RCHO}} + {\text{ H}}_{ 2} {\text{O}} $$

33 $$ {\text{R}}\mathop {\text{C}}\limits^{ \bullet } {\text{HOH }} + {\text{ Fe}}^{ 3+ } \to {\text{RCHO}} + {\text{Fe}}^{ 2+ } + {\text{H}}^{ + } $$

Therefore, during the first protection period alcohols are continuously transformed to the corresponding aldehydes. Actually, the first protection period in case of alcohols is much more developed than that occurring in aldehydes. It is probably possible to assume that the first protection period in presence of alcohols is due to the protective effect occurring during alcohol transformation to aldehydes and aldehydes transformation to the corresponding acids. This probably is confirmed by the fact that the rate constants of OH reaction with alcohols (reactions 26–29) are comparable to the rate constants of OH reactions with the corresponding aldehydes (reactions 14, 15).

The second protection period in case of alcohol additives is similar to that observed in case of aldehyde additives. Valance stability of Fe(II) ions is very probably due to the scavenging effect of the formed organic acids on OH radicals. This occurs until all the formed organic acids are exhausted leading to the restoration of the oxidative effect of OH radicals on Fe(II) ions (reaction 4).

### Radiolysis of Fe(III) acidic solutions in presence of organic additives

**Fig. 4:**
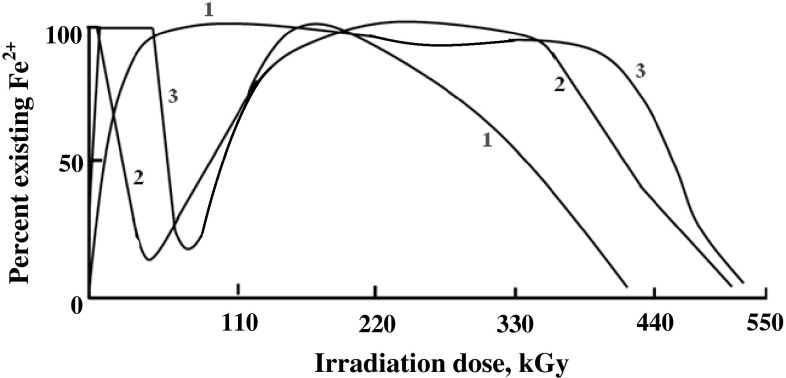
Percent existing Fe^2+^ in γ-irradiated 10^−3^ M Fe^3+^ solutions (0.08 N H_2_SO_4_) containing 16.0 × 10^−3^ M of: *1*—Acetic acid, *2*—Acetaldehyde, *3*—Ethanol

It was interesting to investigate the radiolytic behavior of Fe(III) ions in acidic solutions containing different organic additives. Thus, 10^−3^ M Fe(III) acidic solutions containing organic acids, aldehydes or alcohols, were subjected to extended gamma irradiation. Some representative results are given in Fig. 4. It could be observed that in presence of acetic acid rapid reduction of Fe(III) occurred at the beginning of irradiation until most of these ions were transformed to the divalent state. Then a valence protection period of Fe(II) starts and extends until the acid additive is exhausted whereby a gradual decay of Fe(II) took place until the complete oxidation of iron to the trivalent state.

On using aldehyde or alcohol additives, rapid reduction of iron ions to the divalent state occurred most probably by reaction 8. Then, two protection periods were observed.

In case of aldehyde additive, the first period most probably involves aldehyde protection accompanied with oxidation of the aldehyde to the corresponding acid. When the aldehyde is consumed Fe(II) decays gradually to the trivalent state. This is followed by a reduction stage until iron ions are almost completely reduced to the divalent state whereby the second protection period started and continued until the complete decay of the formed acid.

In case of alcohol additive two protection periods also exists, as in case of aldehyde additive. However, the first protection period is much more developed than in case of aldehyde additives as has been also observed in the Fe(II) systems discussed before. This has been attributed to the consecutive alcohol and aldehyde protection of existing divalent iron in the first protection period. At the end of the first protection period Iron ions are oxidized to the trivalent state. The second protection period started by the gradual reduction of Fe(III) ions to the divalent state. This is followed by a valence stable stage, for Fe(II) until the formed acid is completely exhausted whereby final oxidation of iron(II) occurred. This conforms with the previous discussions on the prevailing reactions in case of Fe(II) systems.

### Effect of concentration of the organic additives and the resultant valence protection of Fe(II) during extended gamma irradiation

**Fig. 5:**
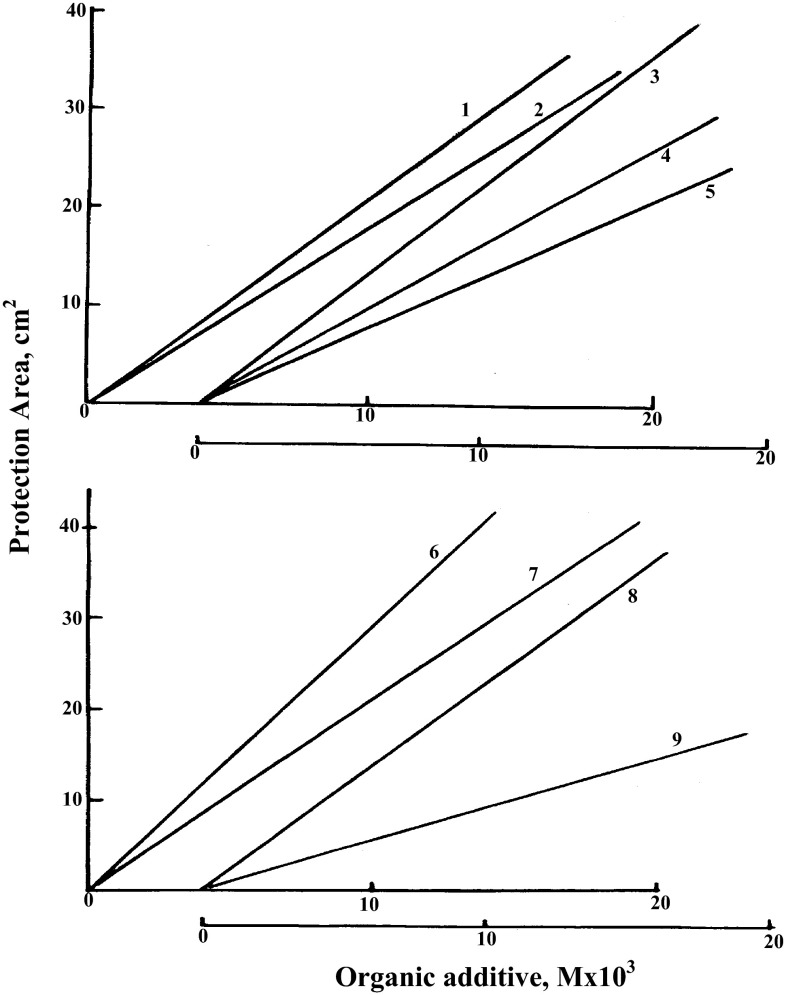
Relationship between the organic additives concentration and the area under the protection curves (in cm^2^) *1*—Acetic acid, *2*—Propionic acid *3*—Acetoldehyde, *4*—Propionaldehyde, *5*—Butyraldehyde, *6*—*n*-Butyl alcohol, *7*—*n*-Propyl alcohol, *8*—Ethyl alcohol, *9*—Methyl alcohol

In order to define the relationship between the amount of the organic additive used and the resultant protection of the Fe(II) ions in the irradiated systems the areas under the actual protection lines, in Figs. 1, 2 and 3 were determined in cm^2^ and were taken as a measure of the occurring protection and were plotted against the corresponding concentrations of the organic additives used. The results are shown in Fig. 5. It is clear that good linear relationships exist between the amount of the organic additive used and the areas under the protection lines. This confirms the relationship between the amount of the protective agent used and the valance protection obtained.

In order to evaluate the overall protection capacity occurring in different systems, the percent of total protection was determined as follows:

$$ \% {\text{ Total protection }} = \, \left( {A_{\text{p}} /A_{\text{t}} } \right) \, \times { 1}00 $$

where *A*_p_ is the area under the protection line and *A*_t_ total area under the 100 % protection line.

The results are given in Table 1. From these results it is clear that on using about 15 m moles of the organic additives per m mole Fe(II) ions, the percent protection is around 70–80 % on using a total dose of about 450 KGys. Lower molar ratios leads to about 60 % protection at a total dose of 150–190 KGys.

**Table 1:** Percent protection of 10^−3 ^M Fe(II) solution during extended gamma irradiation in presence of different organic additives

Additive	Conce. used (mM)	Total dose^a^ (KGy)	Total protection^b^ (%)	Additive	Conce. used (mM)	Total dose^a^ (KGy)	Total protection^b^ (%)
Methanol	14	260	82.9	Ethanol	14	460	76.7
	8	140	67.0		8	310	61.0
	4.8	145	87.1		4.8	178	70.3
	3.2	–	–		3.2	145	72.0
*n*-Proponol	14	460	73.1	*n*-Butonol	14	465	75.0
	8	290	66.4		8	350	78.5
	4.8	185	56.5		4.8	230	76.4
	3.2	175	48.1		3.2	180	60.9
Acetic acid	16	470	83.9	Propionic acid	16	535	72
	8	255	76.7		8	345	86.6
	3.2	165	81.4		3.2	190	71.2
	1.6	95	67.0		1.6	100	53.9
Acetaldehyde	16	515	88.9	Propionaldehyde	16	430	75.6
	8	300	85.4		8	225	68.3
	3.2	167	51.7		3.2	160	54.2
	1.6	115	71.2		1.6	125	47.3
Butyraldehyde	16	415	69.97				
	8	265	64.7				
	3.2	185	67.3				
	1.6	125	59.5				

^a^Total absorbed dose till the complete decay of the used amount of additive

^b^Percent taken as a measure of actual protection

## Conclusions

1. Organic acids, aldehydes and alcohols can effectively protect divalent iron ions against oxidation under the effect of continued gamma irradiation.
2. The extent of protection depends on the type and amount of the additives used.
3. Protection could be attributed to the competition reactions of the divalent iron ions and organic additive for the oxidizing radicals, formed during water radiolysis.
